# Combining PET and Compton imaging with edge-on CZT detectors for enhanced diagnostic capabilities

**DOI:** 10.36922/arnm.3330

**Published:** 2024-06-14

**Authors:** Greyson Shoop, Shiva Abbaszadeh

**Affiliations:** Department of Electrical and Computer Engineering, Baskin School of Engineering, University of California, Santa Cruz, United States of America

**Keywords:** Positron emission tomography/computed tomography, Compton camera, Positron range, Multi-isotope imaging

## Abstract

The key metrics for positron emission tomography (PET) imaging devices include the capability to capture the maximum available amount of annihilation photon information while generating high-quality images of the radiation distribution. This capability carries clinical implications by reducing scanning time for imaging, thus reducing radiation exposure for patients. However, imaging quality is degraded by positron range effects and the non-collinearity of positron annihilation photons. Utilizing an edge-on configuration of cadmium zinc telluride (CZT) detector crystals offers a potential solution to increase PET sensitivity. The high cross-section of CZT and its capacity to detect both 511 keV annihilation gammas and high-energy prompt gammas, along with multiple photon interaction events, contribute to this increased sensitivity. In this study, we propose a dual-panel edge-on CZT detector system comprised of 4 × 4 × 0.5 cm^3^ CZT detectors, with panel dimensions of 20 × 15 cm^2^ and a thickness of 4 cm. In this study, we demonstrate the increased sensitivity of our imaging system due to the detection of the Compton kinematics of high-energy gammas originating from prompt-gamma-emitting isotopes. This was achieved using Monte Carlo simulations of a prompt-gamma-emitting isotope,^72^As, with mean positron ranges >3 mm. Our system’s dynamic energy range, capable of detecting gammas up to 1.2 MeV, allows it to operate in a dual-mode fashion as both a Compton camera (CC) and standard PET. By presenting reconstructions of ^72^As, we highlight the absence of positron range effects in CC reconstructions compared to PET reconstructions. In addition, we evaluate the system’s increased sensitivity resulting from its ability to detect high-energy prompt gammas.

## 1. Introduction

**Figure 1.**
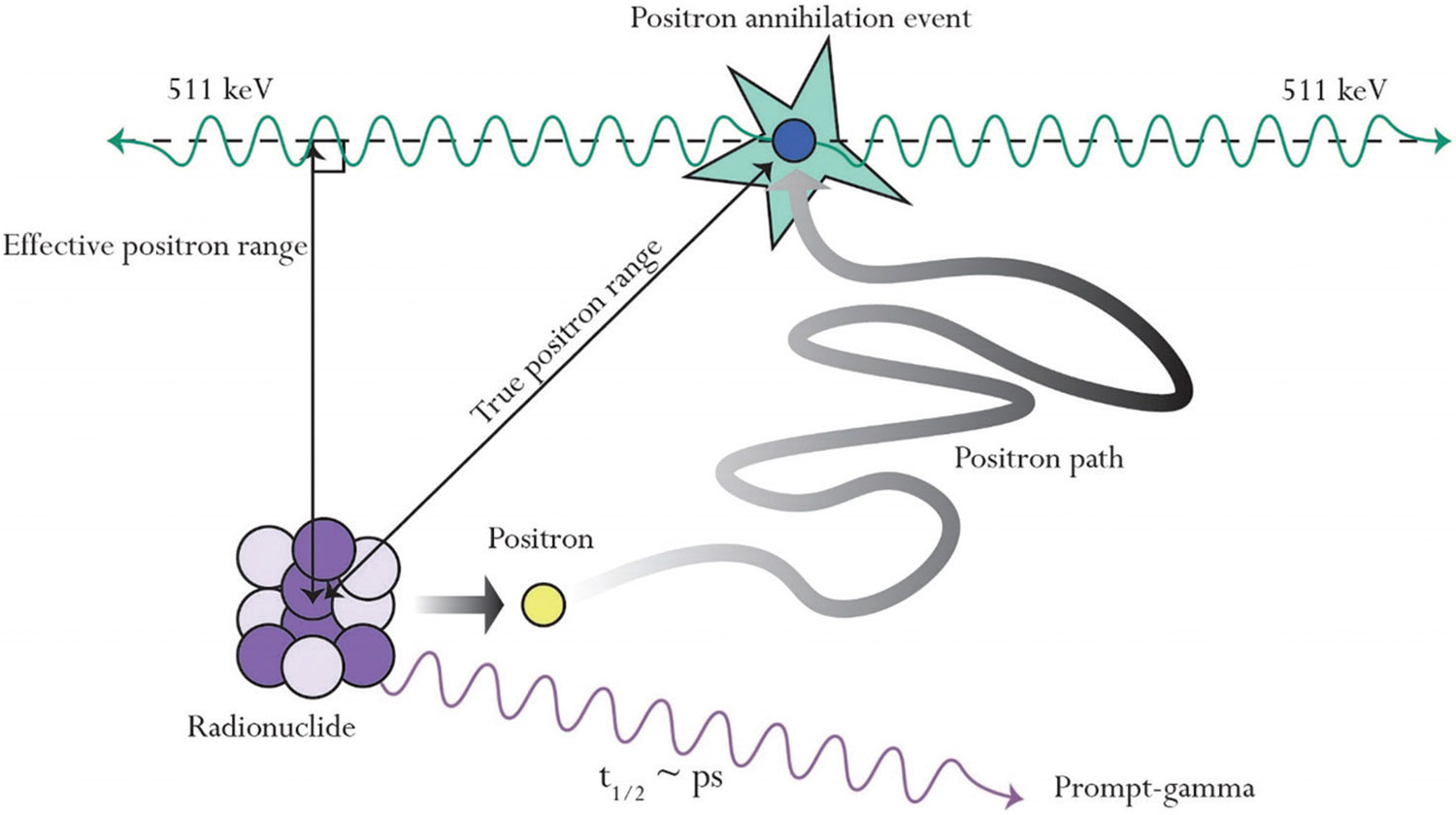
Depiction of positron decay for non-pure positron emitters. Annihilation photons represent the position of positron annihilation position and not the radionuclide position.

The increasing availability of radionuclides for radiopharmaceuticals has positioned targeted radionuclide therapy (TRT) as a viable option for various cancer treatments, which has sparked significant interest in radiotheranostics, a field that combines nuclear imaging with TRT to enable simultaneous non-invasive *in vivo* treatment and visualization of cancerous tissue.^1-3^ The success of radiotheranostics procedures requires high-resolution imaging and dosimetry of tracers to accurately assess the delivery, dose, and activity of radiotherapeutic agents within the patient. However, this leads to non-idealities in positron emission tomography (PET) imaging as the need for non-pure positron ($\beta^{+}$) emitters (positron emitting nuclides with additional gamma emissions) can cause image degradation due to additional gamma emissions overlapping with the 511 keV annihilation photon energy windows. In addition, as depicted in Figure 1, non-pure $\beta^{+}$ emitters exhibit $\beta^{+}$ range effects where the emitted $\beta^{+}$ particle travels significant distances in patient tissue before annihilation occurs, which can cause uncertainty in the true radiotracer distribution. These effects, rooted in physics, present challenges that pre-clinical scanners have yet to overcome despite their exceptional spatial resolution performance.^4,5^

An example where overcoming these challenges is useful is in prostate-specific membrane antigen radioligand therapy (PSMA-RLT). In clinical settings, PSMA-RLT requires the patient to undergo diagnostic PSMA-PET using radionuclides such as ^18^F and ^68^Ga. These radionuclides bind to PSMA pharmaceuticals in the form of piflufolastat ^18^F (^18^F-DCFPyL) and ^68^Ga-PSMA-11, demonstrating superior efficacy for prostate cancer diagnosis compared to [^18^F] Fluorodeoxyglucose PET (FDG-PET).^6-8^ Subsequently, targeted radiotherapy follows, employing either β-emitting nuclides such as ^177^Lu or α emitting nuclides like ^225^Ac attached to PSMA-617. However, ^68^Ga, being a non-pure $\beta^{+}$ emitter, exhibits properties that are shown to have lower spatial resolution compared to ^18^F.^9-11^ Due to the large mean $\beta^{+}$ range (3.56 mm) and a high energy prompt gamma of 1.077 MeV of ^68^Ga, the correction of $\beta^{+}$ range effects and the capability to detect and reject high energy gammas are necessary to improve image quality. While research is still in its early stages regarding the efficacy of various PSMA-PET imaging techniques for proper prognosis, PSMA-RLT remains costly, and the limited supply of ^177^Lu and ^225^Ac further complicates the situation. However, solving these challenges would not only reduce costs but also prevent unnecessary procedures and limit radiation exposure to patients.^12,13^

A summary of various non-pure $\beta^{+}$ emitters that have piqued the interest of researchers, detailing their properties, such as positron range (${\beta^{+}}_{\text{ave}}$) and prompt-gamma (γ) energies, is presented in Table 1.^14-16^ Many of these isotopes are of particular interest for multi-isotope imaging and radiotheranostics despite having large positron ranges and multiple prompt-gamma emissions at various energies and intensities. One notable example is ^89^Zr, a non-pure PET emitter that emits high-energy gammas; however, it doesn’t necessarily qualify as a prompt-gamma emitter due to the long half-life of its excited metastable state (t_1/2_ = 16 s).^16-21^ Nonetheless, ^89^Zr emits a 909 keV gamma with a high yield of 99% of positron decay. This characteristic gamma of ^89^Zr allows the possibility of radiolabeling ^89^Zr separately from another PET tracer, such as ^111^In. This facilitates the simultaneous identification of separate antigens in biological tissues.^22^

**Figure 2.**
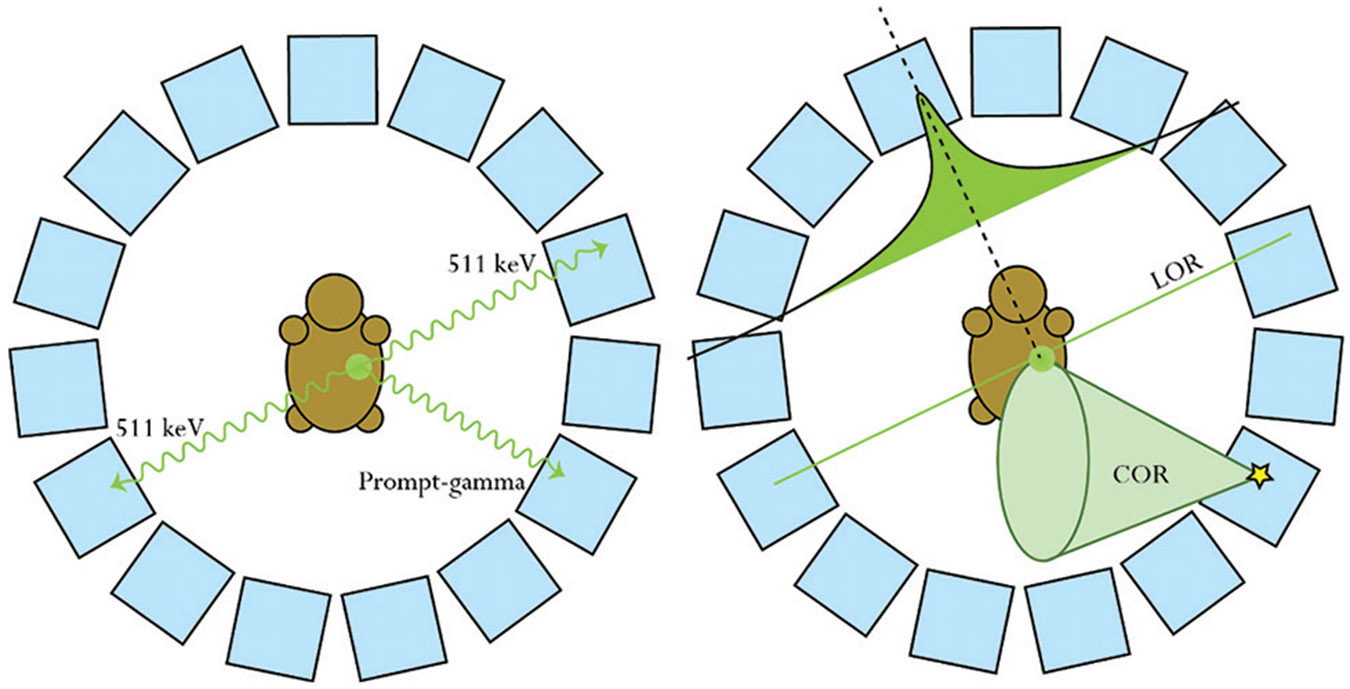
Triple-gamma coincidence techniques for pseudo-time-of-flight image reconstruction Abbreviations: COR: Cone of response; LOR: Line of response.

Recent efforts to tackle these challenges have seen efforts to combine imaging modalities such as PET and Compton camera (CC) imaging to increase spatial resolution through joint reconstruction techniques utilizing high-energy gammas from prompt-gamma emitters.^23-26^ The xenon medical imaging system 2 (XEMIS2) is a small animal system aimed to implement triple-gamma (3-γ) coincidence reconstruction techniques using its liquid xenon time projection chamber technology through a pseudo-time-of-flight (TOF) technique.^25^ Although not the purpose of this work, a potential 3-γ imaging technique is visualized in Figure 2. This method relies on the coincidence detection of a prompt-gamma with annihilation photons. While non-TOF PET back projection techniques will assign equal probability along the line of response (LOR), utilizing LORs with the additional back projection of a cone of response (COR) created by detecting the Compton scattering of higher energy prompt-gammas, it is then possible to localize the source distribution to a smaller segment of the LOR through the LOR-COR intersection.

Addressing the engineering challenge of creating systems for the simultaneous detection of LOR and COR information requires the selection of a scattering detector material with high energy resolution and spatial resolution, as well as the development of an electronic readout scheme that can operate in conjunction with the PET detector layer. Traditionally, in CC systems, two layers of detectors are necessary to obtain CORs, known as the scattering and absorption layers.^27^ The whole gamma imaging system adopts this dual-detector layer approach to induce the scattering of high-energy gammas for detection in coincidence with 511 keV annihilation photons.^23,24^ However, this method presents drawbacks in terms of hardware and electronics complexity for synchronizing two separate devices. In addition, this approach can be costly to implement, as it requires constructing a second detector to be inserted between the radiation source and the PET device.

We propose a dedicated head and neck dual-panel system with an edge-on orientation of detectors, which builds upon extensive research in cross-strip pixelized cadmium zinc telluride (CZT) detectors for small animals and head and neck PET imaging.^28-42^ The CZT detectors in our system are 40 × 40 × 5 mm crystals arranged in an edge-on orientation. The edge-on orientation allows a 4 cm thickness of CZT with a density of 5.78 g cm^−3^ (Z_CZT_ = 48.2), enabling attenuation of high-energy gammas with energy resolution as low as 5%.^41^ Notably, the energy resolution of CZT differentiates itself from other comparable pre-clinical scanners that utilize common scintillation crystals, offering energy resolutions as good as 14%.^43,44^ High-energy resolution crystals are crucial for capturing the Compton scattering of gammas, as the angular resolution depends on energy. The cross-strip electrode design reduces the number of channels for a pixelated detector from n^2^ to 2n channels, with the capability of providing spatial resolutions of up to 1 × 5 mm^2^ in the x-y plane and 1 mm in the z-direction.^31^

Our system offers advantages over conventional hybrid systems in terms of hardware simplicity by eliminating the need for synchronization of separate scattering and absorption layer detector electronics. In theory, this approach could reduce the cost of designing and constructing separate scattering and absorption layer detectors by implementing a single-layer CZT design to capture scattering and absorption interactions. This is made possible by our detectors’ ability to resolve multiple photon interaction events (MIPEs) in the cross-strip design of CZT crystals, in which algorithms have been developed to pair separately, detected intra-crystal scattered MIPE.^31^ Thus, simplifying the problem of implementing a separate or combined imaging modality of PET and CC in software components. Users can then select between separate PET mode, CC mode, or joint PET-CC mode based on the application-whether standard PET, multi-isotope, or triple gamma coincidence imaging. These findings build upon previous work that quantified increases in sensitivity of similar single-layer CZT detector systems for dual PET-CC imaging purposes,^45^ making a significant advancement as no CC reconstruction had been provided until this point.

## 2. Methods

The study utilized Monte Carlo simulation of the dual-panel CZT PET detector imaging system, employing the well-established Geant4 application for tomography emission (GATE) software.^46,47^ The simulated isotope, ^72^As, with its large positron range (5.19 mm), enables comparison of PET and CC reconstruction methods. Furthermore, its prompt-gamma emission (834 keV at 81%) allows us to demonstrate increased system sensitivity by detecting scattering for COR projection data. Reconstruction was performed using ground truth information from the Monte Carlo simulation, i.e., discarding random and scatter coincidences from prompt-gamma down scattering. In addition, PET and CC reconstruction were performed without energy, time, or spatial blurring, demonstrating the best-case scenarios for the performance of PET and CC modalities within the system. Thus, no regularization or filters were applied in our MLEM PET and CC image reconstruction methods. To account for reported energy resolutions of 5.85% and 4.40% at 511 keV and 622 keV, respectively, for flexible circuit-bonded cross-strip CZT detectors, we introduced a 51 keV energy uncertainty to CC projection data.^41^

### 2.1. System geometry

**Figure 3.**
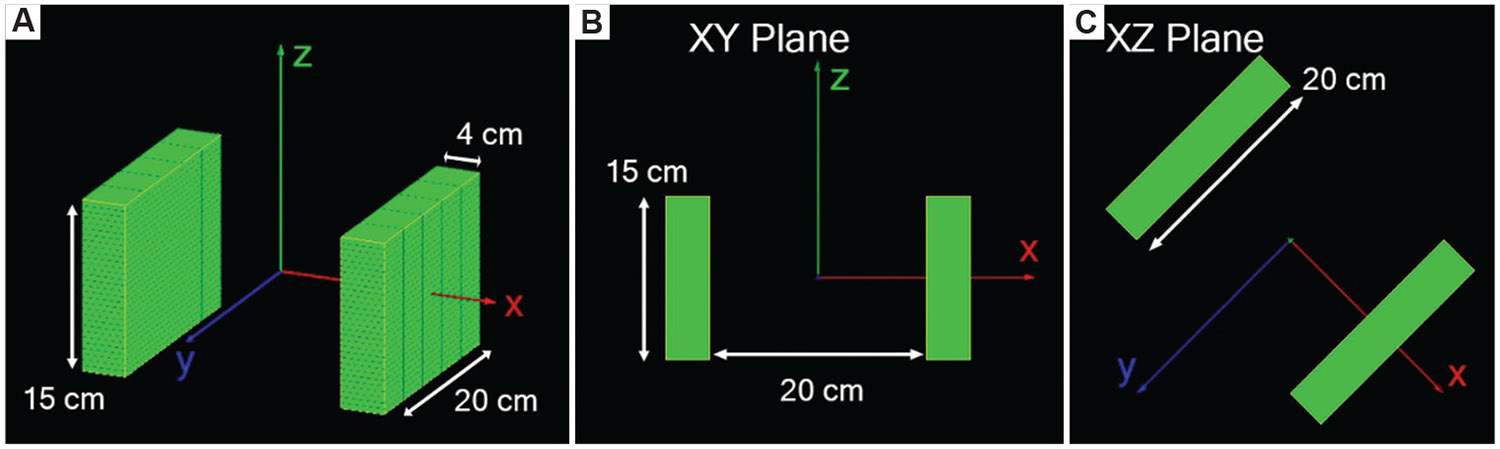
System geometry visualized in Geant4 application for tomography emission. (A) View of dual panel system with dimensions and cartesian coordinate axis. (B) View of dual panel system along y-z plane. (C) View of dual panel system along x-z plane.

The dual-panel CZT PET detector, as constructed in GATE, is presented in Figure 3. It consists of 4 × 4 × 0.5 cm^3^ CZT detector crystals. Each panel comprises 150 CZT detector crystals arranged in five columns of 30 edge-on stacked CZT detectors. The panels boast a detector surface of 20 × 15 cm^2^, with a thickness of 4 cm and a distance of 20 cm between the faces of the two panels.

### 2.2. Radioisotope definitions

In our simulations using GATE, radioisotopes were specified with the ion source definitions, including atomic number (Z), atomic weight (A), ionic charge (Q), and excitation energy (E). This configuration enables the simulation of radioactive decay and atomic de-excitation physics, enabling the emission of positrons and their kinematics, as well as positron annihilation. In addition, all electromagnetic interaction physics involving annihilation photons and prompt gammas with the CZT detector crystals are specified using the em_standard_opt4 physics list provided in GATE.

**Figure 4.**
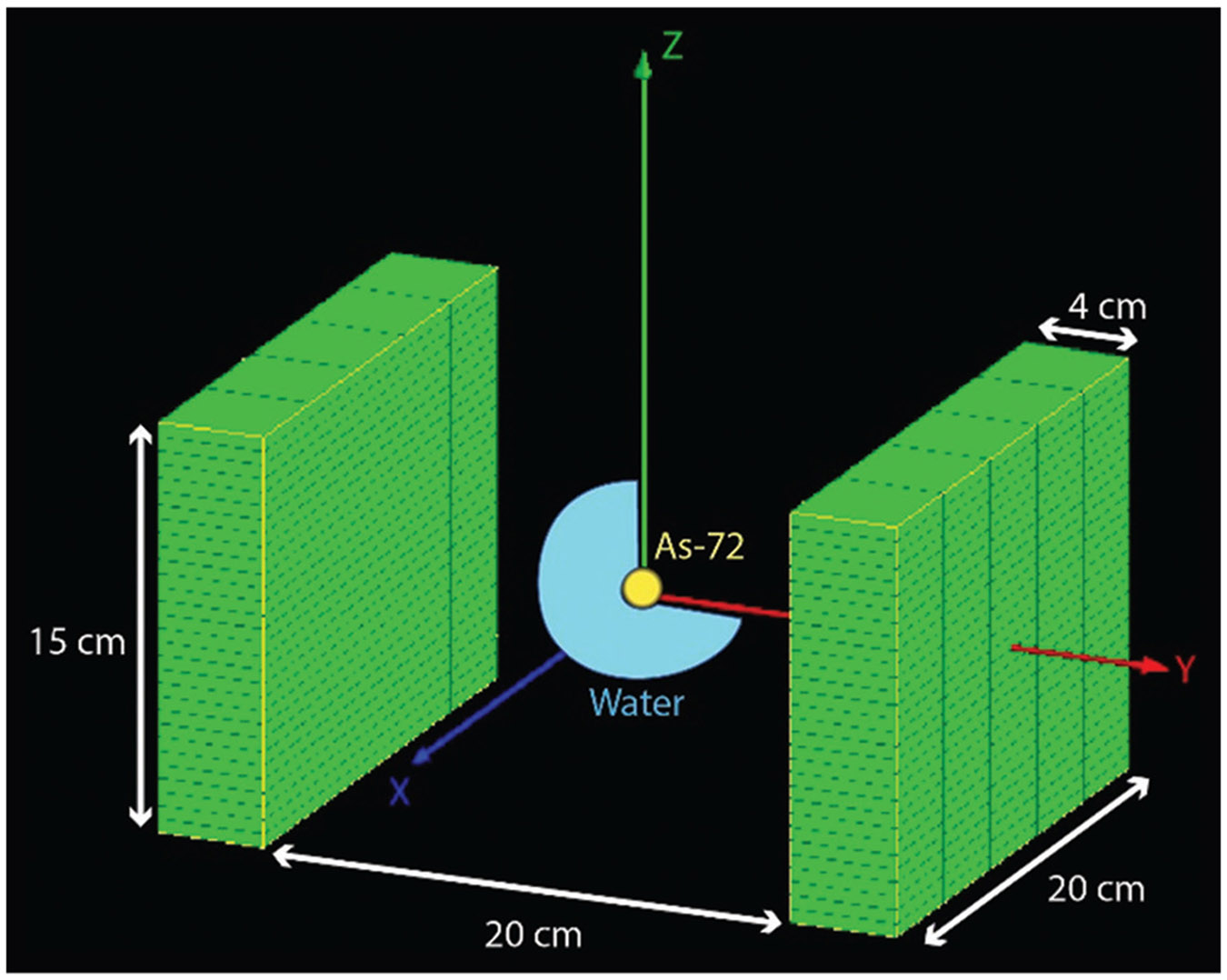
Experiment visualization. A 0.1 mm radius ^72^As spherical point source with 2 MBq of activity is placed at the origin within a spherical water phantom of 2 cm diameter. The source is located at (0, 0, 0) mm central to the orientation of the dual panel system.

In this study, we simulated ^72^As with an activity of 2 MBq over a 1 s acquisition time. The visualization of the experiment in GATE is depicted in Figure 4. The source, represented as a 0.1 mm radius sphere, is positioned centrally to the panels at the origin of a Cartesian coordinate space (x, y, z) specified at (0, 0, 0). To demonstrate positron range and radiotracer behavior in soft-tissue equivalent material, the source was placed centrally within a spherical water phantom of 2 cm diameter.

### 2.3. Image reconstruction

We performed PET image reconstruction utilizing an in-house list-mode maximum likelihood expectation maximization (LM-MLEM) iterative reconstruction code, implemented in the compute unified device architecture (CUDA) software. This method follows the standard formulation of LM-MLEM as described by Equation I.^48,49^

(I) $$f_{j}^{\left(k+1\right)}=\frac{f_{j}^{\left(k\right)}}{s_{j}}\sum_{i}a_{ij}\left(\frac{p_{i}}{\sum_{j}a_{ij}f_{j}^{\left(k\right)}}\right)$$

CC image reconstruction follows a similar manner, employing an open-source LM-MLEM iterative reconstruction CUDA code, formulated as in Equation I. The initial image was initialized to uniformity, represented by $f_{j}^{\left(0\right)}=1$. The system matrix, $a_{ij}$ was constructed in our PET reconstruction based on orthogonal distance-based ray-tracer (OD-RT) projectors and a fixed Gaussian kernel for the tube of response (TOR).^50^ In the CC reconstruction, the system matrix was constructed based on a ray-tracing method where the surfaces of the cone projections are sampled as a set of line samples with energy-based Gaussian kernels for the volume of response (VOR).^51,52^ We assumed uniformity for the detector sensitivity in both methods, denoted as $s_{j}=1$. Since LM is implemented for both reconstructions, only the captured projections were considered, set as $p_{i}=1$. Implementation of angular blurring in the form of energy resolution and doppler broadening was taken into account in the system matrix construction of the CC reconstruction.^53,54^ The 3D image reconstruction was done on a 40 × 40 × 40 voxel grid constituting the image space, with voxel dimensions of 1 × 1 × 1 mm^3^.

To prepare LM data for both CC and PET image reconstruction, we utilized two separate Python scripts to parse *a priori* GATE hits output files for each simulation. The CC LM format is a text file where each row represents projection data for the detection of prompt gammas. The columns include the x, y, and z coordinates of the Compton scattering position of a prompt-gamma, along with the energy transferred, as well as the x, y, and z coordinates of the subsequent photoelectric absorption position and the associated energy transferred. Thus, each row represents COR information for a detected prompt-gamma, considering only the sequence of a Compton scattering followed by a photoelectric absorption event for the CORs. On the other hand, the PET LM format is a text file where each row represents the projection data from the detection of two annihilation photon pairs, i.e., the LORs. The columns describe the x, y, and z coordinates of both annihilation photons.

### 2.4. Statistical analysis

The statistics of particle-matter interactions of the photons within the detector system in the Monte Carlo simulation are output and analyzed using the ROOT data analysis framework.^55^

Python code was written to extract the estimation of the voxels and create 2D histograms of the estimated source activity in the coronal, sagittal, and transverse planes. In addition, this code computes the full width at half-maximum (FWHM) of the reconstructed normalized activity profiles along all three axes using Gaussian fitting, as described in Equation II,

(II) $$p(x)=ae^{-\frac{1}{2}{\left(\frac{x-\mu }{\sigma }\right)}^{2}};a=\frac{1}{\sigma \sqrt{2\pi }}$$

where p(x) represents the probability density function along the normalized activity profile spanned by x with mean μ and standard deviation σ. The FWHM and FWTM were computed using Equations III and IV.


(III) $$FWHM=2\sigma \sqrt{2ln(2)}=2.35\sigma$$

(IV) $$\text{FWTM}=2\sigma \sqrt{2\;\text{ln}(10)}=4.29\sigma$$

When computing fits for distributions provided by PET reconstruction, Lorentzian fits of the form (Equation V) were employed to better accommodate the positron range effects,

(V) $$L(x)=A\frac{\Gamma }{{\left(x-x_{0}\right)}^{2}+\Gamma^{2}}$$

where L(x) represents the probability density function along the normalized activity profile spanned by x with peak center $x_{0}$ and half-width at half-maximum (HWHM) Γ. The FWHM is therefore equal to 2Γ while the FWTM is computed as $2\Gamma \sqrt{9}$.

## 3. Results

### 3.1. Sensitivity

**Figure 5.**
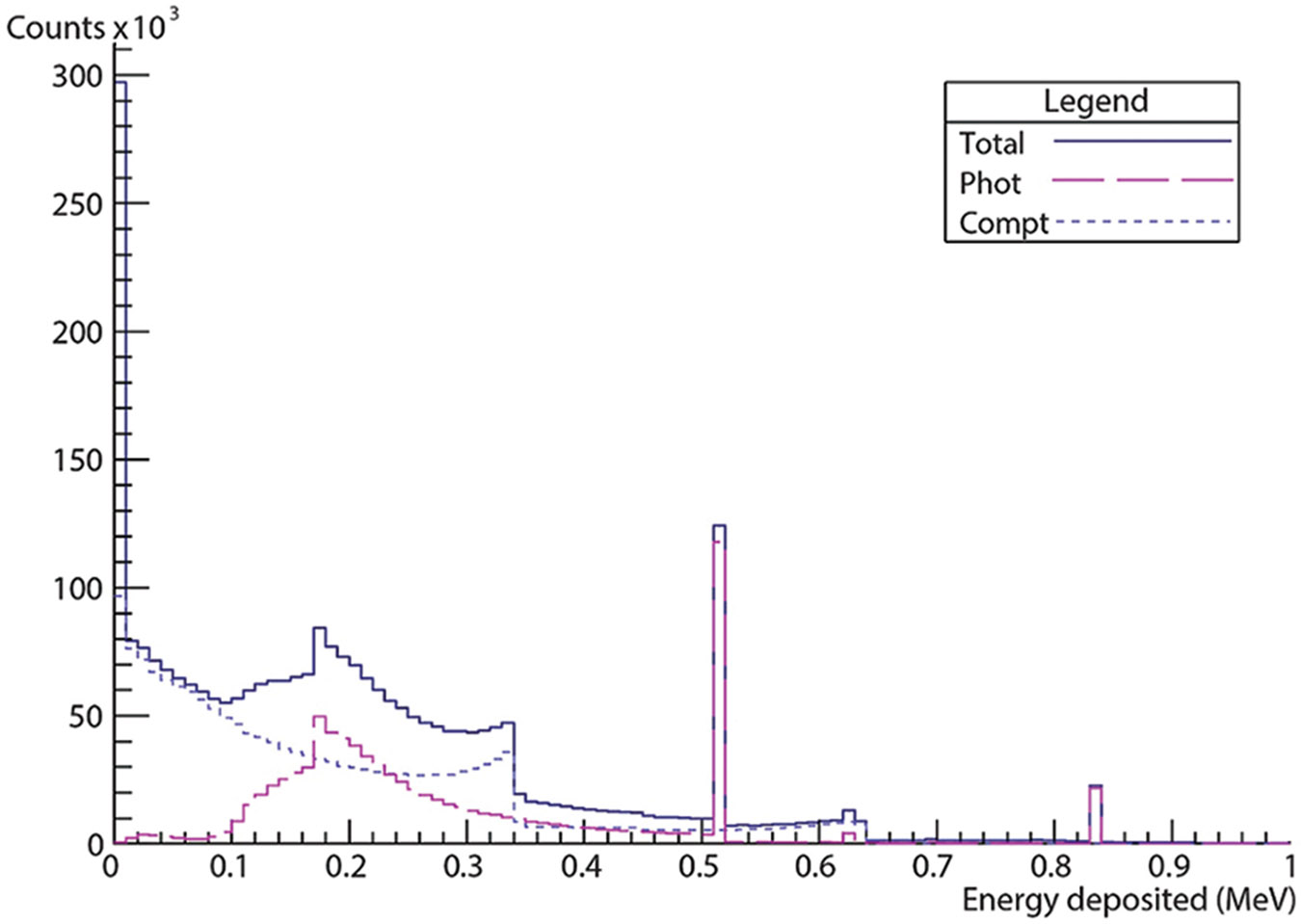
Energy deposition histogram from Geant4 application for tomography emission simulation. Energy deposition spectrum from cadmium zinc telluride detector from simulation of a 2 MBq ^72^As point source with photoelectric events (phot) and Compton scattering events (compt) plotted separately.

An energy histogram of what the dual-panel CZT system can detect is presented in Figure 5, obtained from ROOT output containing *a priori* information on electromagnetic interactions within the CZT crystals. The solid marker represents the total energy spectrum comprising photoelectric interactions, Compton scattering, and Rayleigh scatterings. The large pink, dashed marker represents the energy spectrum of photoelectric interactions as detected by the CZT detector, while the small, dashed marker represents the energy spectrum of Compton scattering interactions as detected by the CZT detector. We identified photoelectric peaks at 511 keV and 834 keV, attributed to the annihilation photons and the prompt-gamma energy from ^72^As, noting that the yield of the 834 keV gamma was approximately 81%. In addition to the drop in the photoelectric cross-section for CZT for higher energy gammas, the peak appears significantly smaller than that of 511 keV. Considering that over twice as many 511 keV photons are emitted compared to 834 keV gammas and the smaller cross-section for photoelectric absorption at 834 keV compared to 511 keV, the less pronounced peak at 834 keV is understandable. In addition, part of the Compton continuum of scattering energies above 511 keV and below 834 keV, as well as the observation that the Compton continuum of scattering energies originating from the prompt-gamma falls through and below the annihilation photon energy window around 511 keV. This would explain the distribution of photoelectric absorption below the Compton shelf of the 511 keV photons at 340 keV, where the cross-section of photoelectric absorption is significantly higher for CZT.

### 3.2. Image reconstruction and comparison

**Figure 6.**
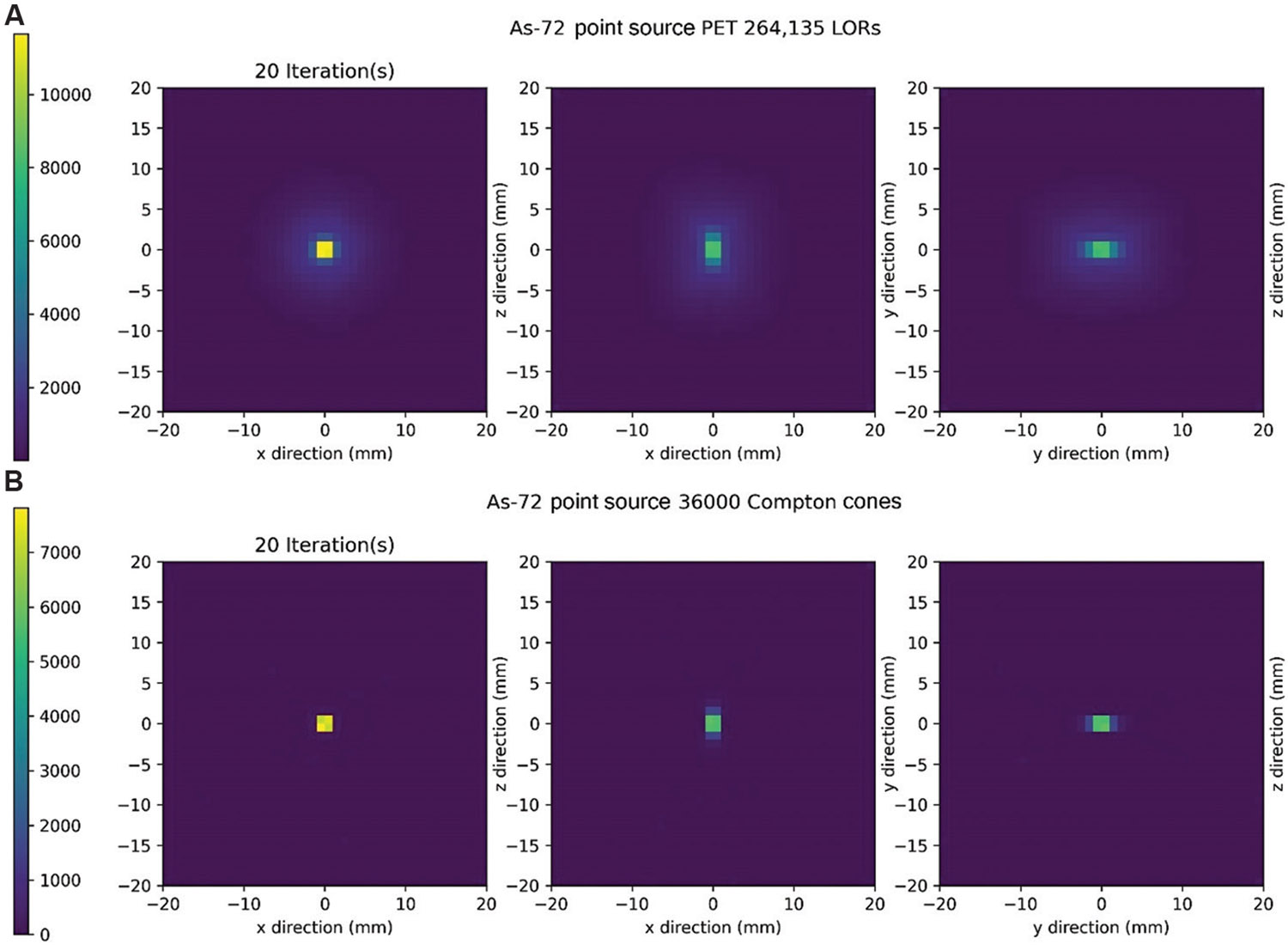
Maximum likelihood expectation maximization reconstruction after 20 iterations. (A) Positron emission tomography reconstructions along all imaging planes. (B) Compton camera (CC) reconstructions along all imaging planes were performed with 1 keV of energy blurring. Abbreviation: LOR: Line of response.

The results of PET and CC image reconstruction following 20, 800, and 3500 iterations of MLEM are presented. The PET and CC image reconstructions after 20 iterations are displayed in Figures 6A and B, respectively. Notably, no energy blurring was applied in the PET reconstruction, while an equivalent of 1 keV energy blurring was applied in the CC reconstruction. The execution time for 20 iterations of MLEM was 1.73 s for PET and 2.97 s for CC. The positron range effects of the ^72^As isotope are present in the PET reconstruction, while the CC reconstruction appears relatively artifact-free aside from the limited artifact smearing in the y direction.

**Figure 7.**
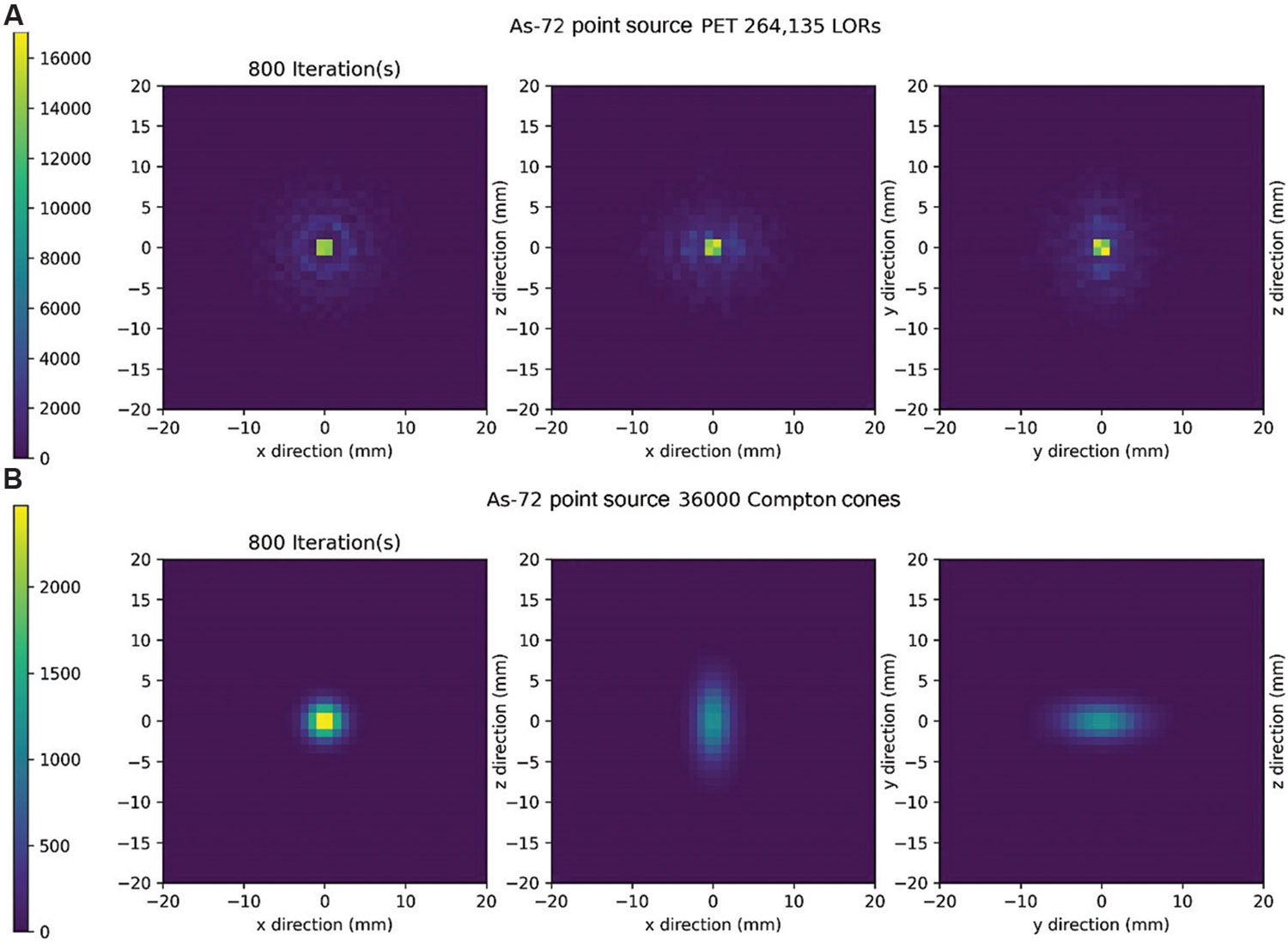
Maximum likelihood expectation maximization reconstruction after 800 iterations. (A) Positron emission tomography reconstructions along all imaging planes. (B) Compton camera reconstructions along all imaging planes were performed with 51 keV of energy blurring. Abbreviation: LOR: Line of response.

Reconstruction after 800 iterations of MLEM is displayed in Figure 7A for PET and in Figure 7B for CC. In this reconstruction, energy blurring equivalent to 51 keV was applied in the CC reconstruction. The time taken for 800 iterations of MLEM was 64.00 s for PET and 25.81 s for CC. The introduction of energy blurring significantly impacted the CC reconstruction, necessitating more iterations to achieve comparable results to PET. The lack of smoothness in the blurring observed in the PET reconstruction compared to CC demonstrates the stochastic nature of the positron range and energy transfer. In addition, we note that image contrast is poorer in the xy and yz planes compared to the xz plane, which parallels the detector panel faces.

**Figure 8.**
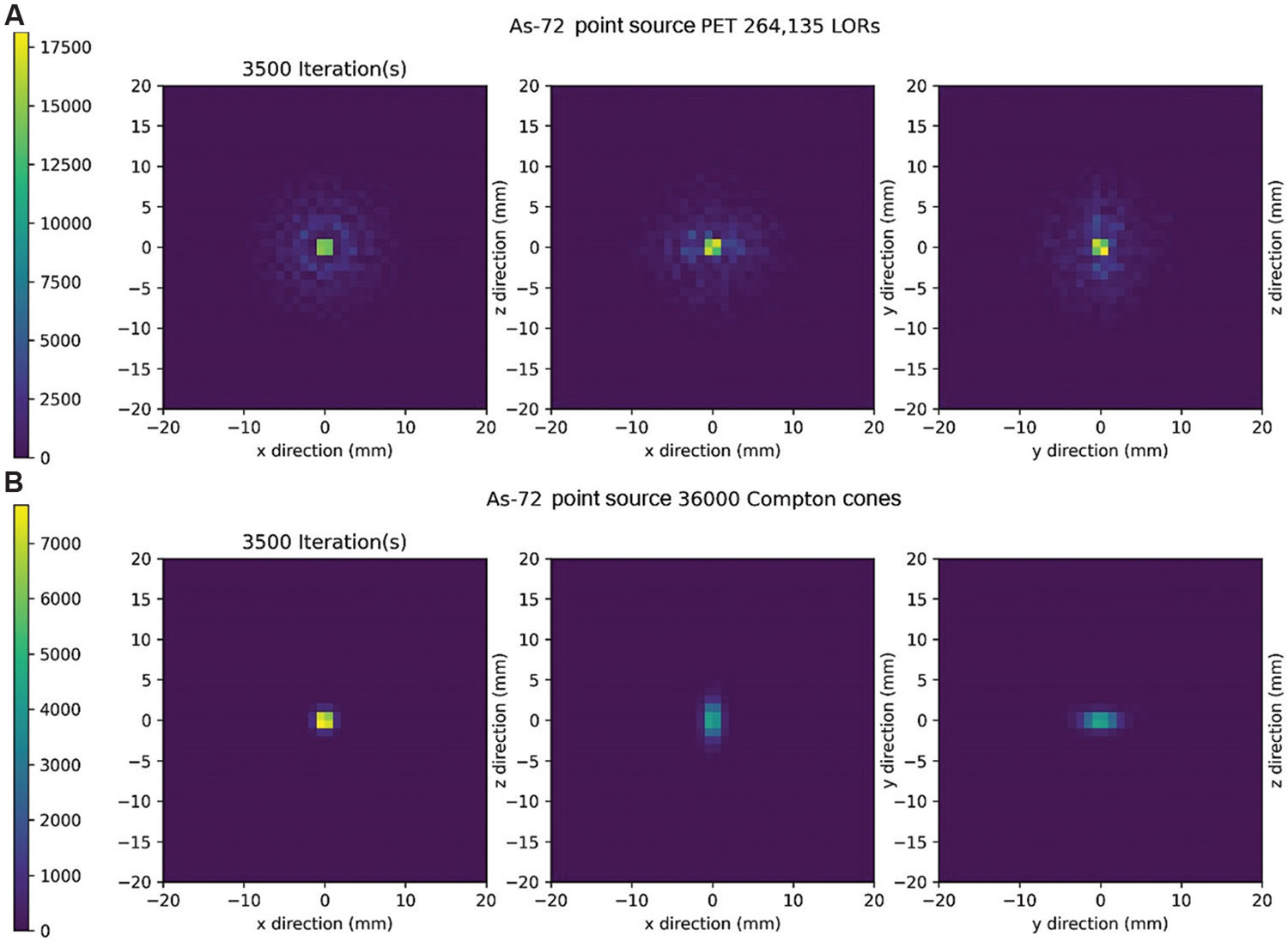
Maximum likelihood expectation maximization reconstruction after 3500 iterations. (A) Positron emission tomography (PET) reconstructions along all imaging planes. (B) Compton camera reconstructions along all imaging planes were performed with 51 keV of energy blurring. Abbreviation: LOR: Line of response.

Finally, after 3500 iterations, Figure 8A depicts the PET reconstruction, and Figure 8B displays the CC reconstruction. The time required for 3500 iterations was 287.30 s for PET and 62.76 s for CC. Notably, after 3500 iterations, the CC reconstruction exhibits significantly improved image contrast and demonstrates precise localization of the source position free from positron range artifacts.

### 3.3. Evaluation of activity distribution

**Figure 9.**
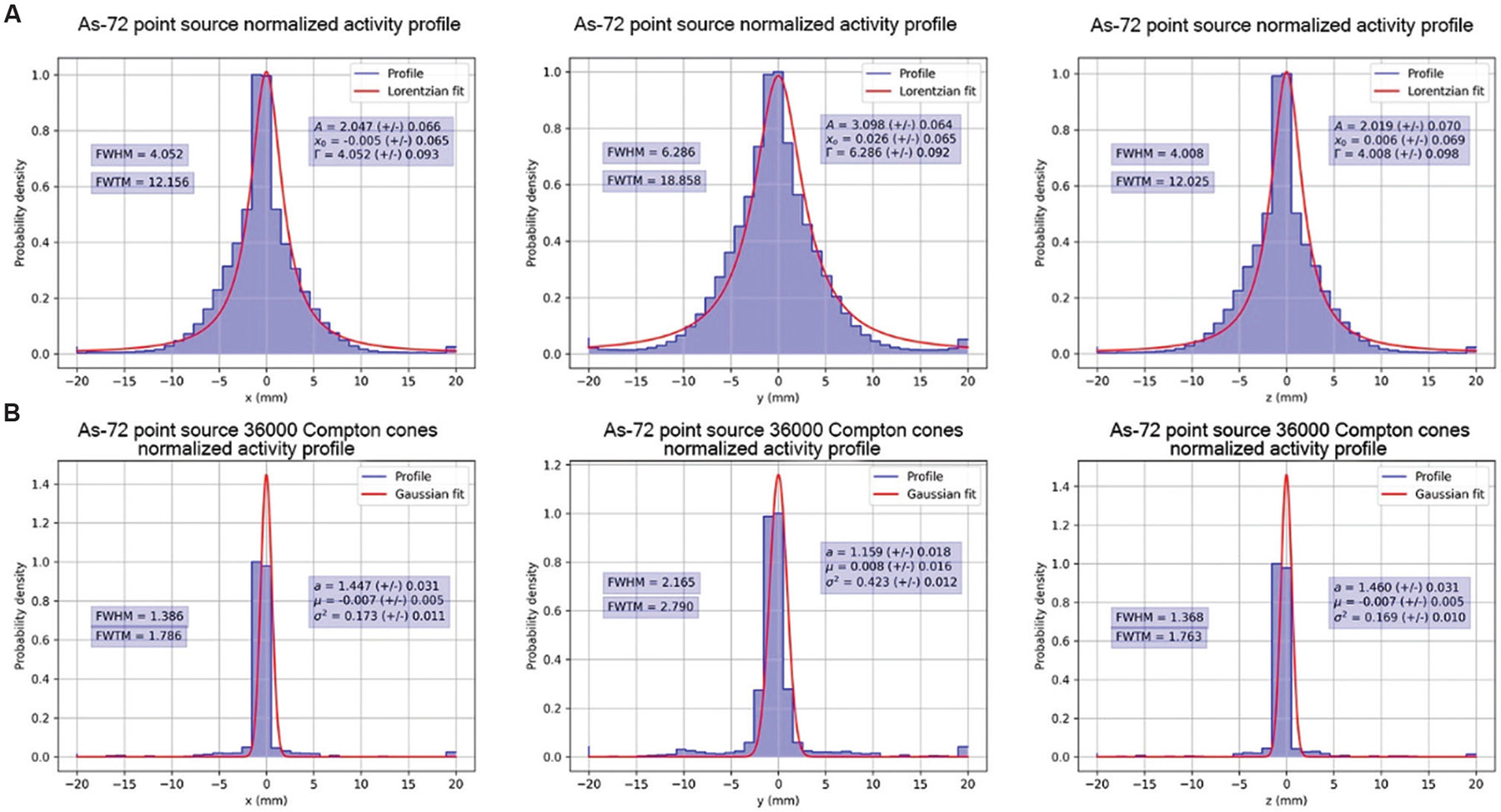
Normalized activity profiles of Figure 6 with Gaussian fits after 20 iterations of MLEM. (A) Top row, normalized activity profiles in the x, y, and z directions of positron emission tomography MLEM. (B) Bottom row, normalized activity profiles in the x, y, and z directions of Compton camera MLEM. Abbreviation: MLEM: Maximum likelihood expectation maximization.

**Figure 10.**
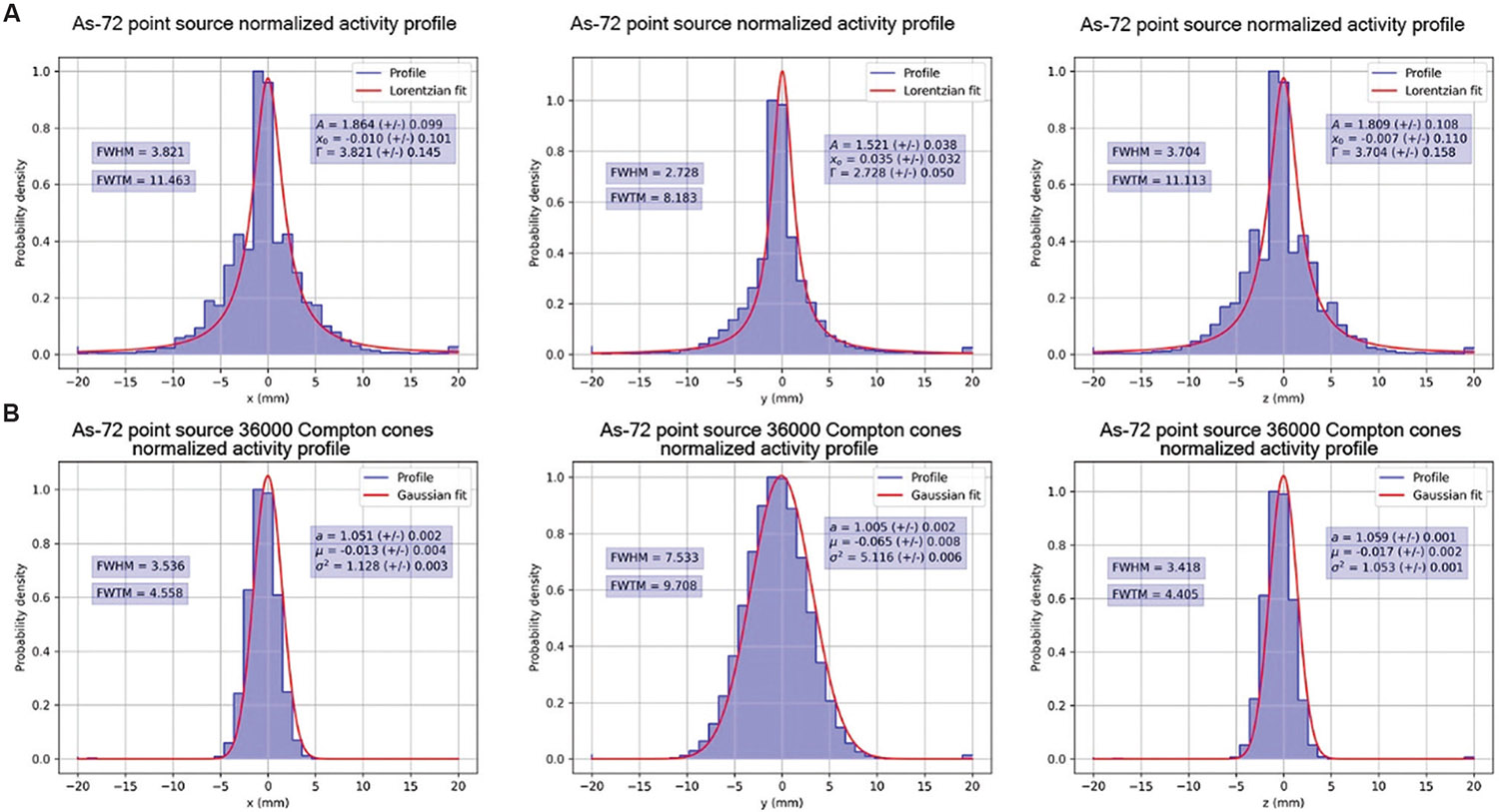
Normalized activity profiles of Figure 7 with Gaussian fits after 800 iterations of MLEM. (A) Top row, normalized activity profiles in the x, y, and z directions of positron emission tomography MLEM. (B) Bottom row, normalized activity profiles in the x, y, and z directions of Compton camera MLEM. Abbreviation: MLEM: Maximum likelihood expectation maximization.

**Figure 11.**
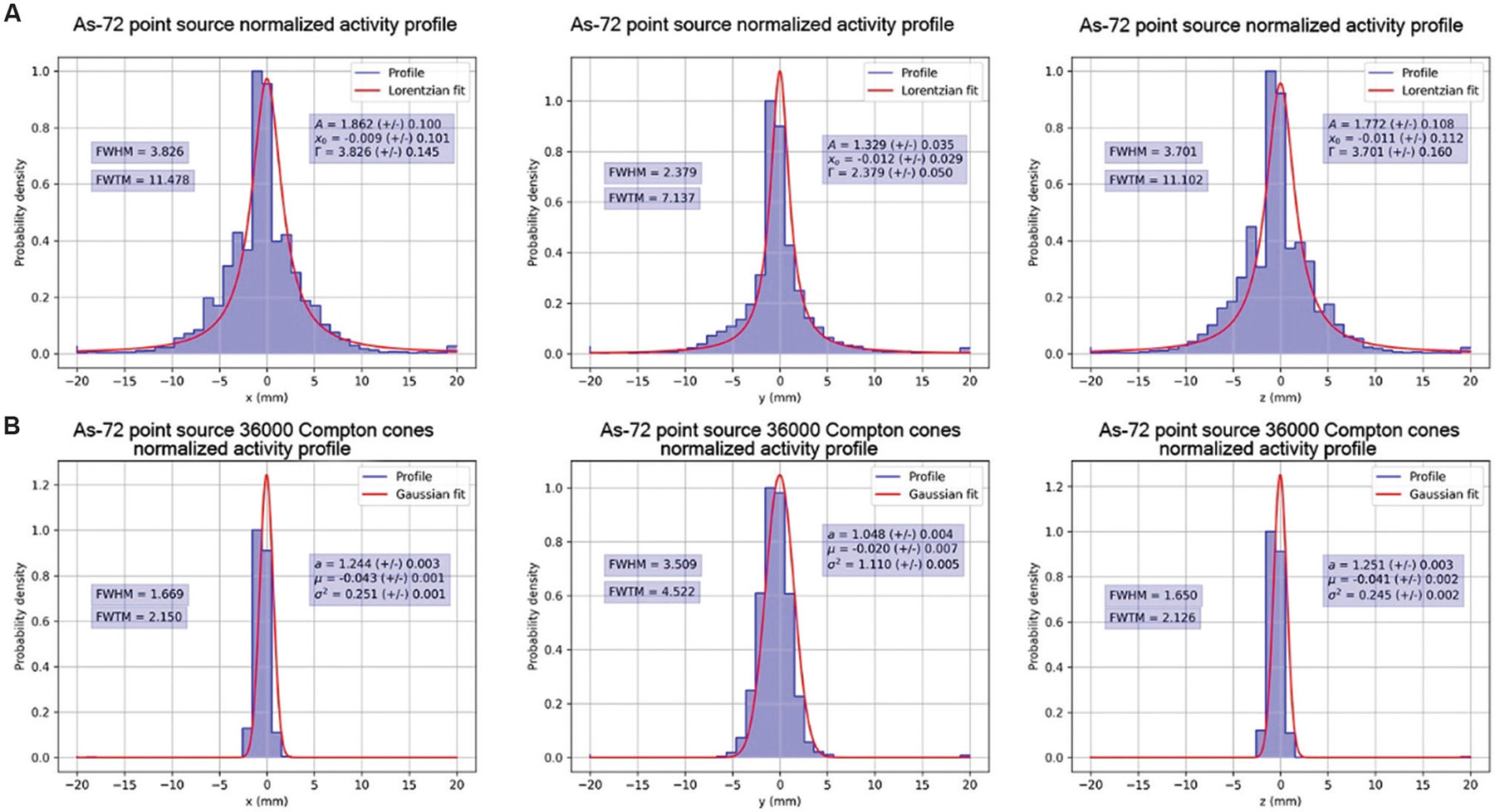
Normalized activity profiles of Figure 8 with Gaussian fits after 3500 iterations of MLEM. (A) Top row, normalized activity profiles in the x, y, and z directions of positron emission tomography MLEM. (B) Bottom row, normalized activity profiles in the x, y, and z directions of Compton camera MLEM. Abbreviation: MLEM: Maximum likelihood expectation maximization.

Normalized activity profiles are presented with computed Gaussian and Lorentzian fits and their associated FWHM and FWTM along the x, y, and z directions of the reconstructed images after 20, 800, and 3500 iterations of MLEM in Figures 9-11, respectively. Figures 9A, 10A, and 11A show the normalized activity profiles of the PET reconstructed images, and Figures 9B, 10B, and 11B show the normalized activity profile of the CC reconstructed images.

The computed FWHM values are summarized in Table 2, and the computed FWTM values are summarized in Table 3. The computed FWHM values in the x and z directions of the PET reconstruction (3.701 – 4.025 mm) show little to no improvement with iteration number and are a consequence of the positron range blur. However, the computed FWHM of CC reconstruction with 51 keV of energy blur in the x (1.669 mm) and z directions (1.650 mm) show that under ideal conditions, the energy resolution of CZT crystals allows for correction of positron range effects in the x and z directions.

## 4. Discussion

The results from the GATE simulation demonstrate an increase in sensitivity for the detection of the Compton kinematics of high-energy prompt-gammas that enter the CZT crystals. This enhancement can be attributed to the high Compton scattering cross section of CZT and the 4 cm edge-on detector thickness, which allows for the subsequent photoelectric detection of Compton scattered gammas originating from prompt gammas. This permits our dual-panel CZT system to gather COR projection data, which is used for CC image reconstruction free of positron range effects. The results of this study do not directly lead to an increased PET sensitivity for this system; however, in potential joint PET-CC reconstruction algorithms, if the subset of COR-LOR coincidences, such as those referenced in Figure 3, is used to supplement PET reconstruction of the superset of LORs, then this would be seen as an increase in overall system sensitivity.

Moreover, CC reconstruction using our dual-panel system demonstrates the capability to estimate source locations free of positron-range artifacts in the xz plane parallel to the detector faces. Although the number of iterations of MLEM needed to provide 1 mm FWHM measurements in the x and z directions significantly increases when taking CZT energy resolution (5%) into account, this provides promising results that CC information retained by the system can be leveraged to provide improved CC-informed PET reconstruction. Future work must be carried out in the implementation of existing concepts in the joint reconstruction of LOR and COR data or the development of system-specific joint reconstruction.^16,25^ It’s important to note that the resolution for both PET and CC was degraded in the y direction of our scanner. This degradation in the y direction is typical for a dual-panel scanner and is a limitation for overcoming positron range corrections in this dimension.^56^ Either a cylindrical geometry must be used in place of a dual-panel geometry or progress in limited-angle artifact correction must be made for implementation in joint PET-CC reconstructions.

Furthermore, the system’s capability to detect Compton scattering not only from prompt gammas but also from annihilation photons holds significant implications for future work in random rejection. With growing interest in the experimental validation of quantum entanglement of annihilation photons,^57-61^ there’s potential for substantial advancements in rejecting random, particularly when dealing with non-pure $\beta^{+}$ emitters like ^72^As, which emit high-energy gammas that contaminate the 511 keV PET energy window.

## 5. Conclusion

We conclude that our dual-panel CZT system is a simple and cost-effective detector with the ability to leverage prompt gamma for hybrid PET and CC imaging applications. This functionality opens avenues for positron range correction, random rejection, and multi-isotope imaging, underscoring the potential impact of our device. With these capabilities, our system is in a strong position to lead the next generation of hybrid imaging PET devices.

## Figures and Tables

**Table 1.** Summary of various non-pure positron-emitting isotope properties and applications

Isotope	Half-life (h)	$\beta^{+}$ yield (%)	${\beta^{+}}_{\text{ave}}$ range (mm)	Prompt γ (keV)	γ yield (%)	Application
^18^F	1.83	97	0.62	NA	NA	Pure PET emitter used in FDG for use in oncology
^68^Ga	1.13	89	3.56	1,077	3.2	Aid for radiotherapy in PSMA-PET
^72^As	26.00	88	5.19	693	8.07	Used as an imager for radiotherapy
				834	81.0	
^89^Zr	18.40	23	1.27	909	99.0	Used in immuno-PET and multi-isotope imaging in head and neck squamous cell carcinoma
^44^Sc	4.04	94	2.46	1,157	99.4	Aid for radiotherapy in PSMA-PET
^124^I	100.32	23	3.37	603	62.9	Used in imaging pharmacokinetics of radiopharmaceuticals
				723	10.4	
				1,691	11.2	

Abbreviations: FDG: ^18^F-fluorodeoxyglucose; PET: Positron emission tomography; PSMA-PET: Prostate-specific membrane antigen positron emission tomography.

**Table 2.** Summary of full width at half-maximum results from normalized activity profiles

Iteration	FWHM (mm)								
	20			800			3500		
	x	y	z	x	y	z	x	y	z
Profile									
PET	4.052	6.286	4.008	3.821	2.728	3.704	3.826	2.379	3.701
CC	1.386	2.165	1.368	3.536	7.533	3.418	1.669	3.509	1.650

Abbreviations: CC: Compton camera; FWHM: Full width at half-maximum; PET: Positron emission tomography.

**Table 3.** Summary of full width at tenth-maximum results from normalized activity profiles

Iteration	FWHM (mm)								
	20			800			3500		
	x	y	z	x	y	z	x	y	z
Profile									
PET	12.156	18.858	12.025	11.463	8.183	11.113	11.478	7.137	11.102
CC	1.786	2.790	1.763	4.558	9.708	4.405	2.150	4.522	2.126

Abbreviations: CC: Compton camera; FWTM: Full width at tenth-maximum; PET: Positron emission tomography.
